# Efficacy of n-3 polyunsaturated fatty acids and feasibility of optimizing preventive strategies in patients at high cardiovascular risk: rationale, design and baseline characteristics of the Rischio and Prevenzione study, a large randomised trial in general practice

**DOI:** 10.1186/1745-6215-11-68

**Published:** 2010-05-28

**Affiliations:** 1 Istituto di Ricerche Farmacologiche "Mario Negri", Via Giuseppe La Masa, 19 20156 Milano, Italy; 2 Consorzio Mario Negri Sud Via Nazionale, 66030 Santa Maria Imbaro, Italy

## Abstract

**Background:**

The optimization of preventive strategies in patients at high risk of cardiovascular events and the evaluation of bottlenecks and limitations of transferring current guidelines to the real world of clinical practice are important limiting steps to cardiovascular prevention. Treatment with n-3 polyunsaturated fatty acids improves prognosis after myocardial infarction, but evidence of this benefit is lacking in patients at high cardiovascular risk, but without a history of myocardial infarction.

**Methods/design:**

Patients were eligible if their general practitioner (GP) considered them at high cardiovascular risk because of a cardiovascular disease other than myocardial infarction, or multiple risk factors (at least four major risk factors in non-diabetic patients and one in diabetics).

Patients were randomly allocated to treatment with n-3 polyunsaturated fatty acids (1 g daily) or placebo in a double-blind study and followed up for five years by their GPs to assess the efficacy of the treatment in preventing cardiovascular mortality (including sudden death) and hospitalization for cardiovascular reasons. The secondary, epidemiological, aim of the study is to assess whether it is feasible to adopt current guidelines in everyday clinical practice, with a view to optimizing all the available preventive strategies in people at high cardiovascular risk.

A nation-wide network of 860 GPs admitted 12,513 patients to the study between February 2004 and March 2007. The mean age was 64 years and 62% were males. Diabetes mellitus plus one or more cardiovascular risk factors was the main inclusion criterion (47%). About 30% of patients were included because of a history of atherosclerotic cardiovascular disease, 21% for four or more risk factors, and less than 1% for other reasons.

**Discussion:**

The Rischio and Prevenzione (R&P) project provides a feasible model to test the efficacy of n-3 polyunsaturated fatty acid therapy in patients at high cardiovascular risk with no history of myocardial infarction, and to assess how to implement recommended preventive strategies in general practice.

**Trial registration:**

ClinicalTrials.gov NCT00317707

## Background

Cardiovascular diseases (CVD) are the leading cause of death in middle-aged and older adults in most European countries. CVD are also an important cause of disability and morbidity and the main economic burden for health care services [1-4].

According to guidelines, patients with established CVD and those with multiple risk factors are at high cardiovascular (CV) risk and are therefore the main target for preventive strategies. These have to be tailored to the level of CV risk rather than aimed "only" at the treatment of individual cardiovascular risk factors [5]. Inadequate control of modifiable risk factors has been documented in various surveys [6-12] so there is considerable potential for improving cardiovascular prevention, especially in everyday clinical practice

While it is easy to see that general practice is a suitable setting for large-scale randomized controlled trials (RCTs) and prospective outcome-oriented studies [13-15], it is still rare to find general practice-based reports in the formulation and enforcement of guidelines for primary care physicians [16]. It is clear that until general practice itself is directly involved in research programs no significant or pertinent changes will ever be proposed and adopted.

Among possible preventive strategies, n-3 polyunsaturated fatty acids of marine origin (n-3 PUFA) are the newest and most promising. N-3 PUFA have been evaluated in pharmacological studies for their antithrombotic and anti-atherosclerotic effects, and their positive action on arrhythmias [17,18] has attracted a lot of attention.

After a pilot study to assess the feasibility of a large intervention study in the setting of general practice [10,19], the Rischio&Prevenzione (R&P) Study was launched in 2004. The project was designed to follow a cohort of patients at high CVD risk but with no history of myocardial infarction to test the efficacy of n-3 PUFA therapy, on the top of the other recommended preventive strategies (including lifestyle intervention and pharmacological treatments) aimed at optimizing the cardiovascular risk profile.

### Rationale of the n-3 PUFA hypothesis

The cardioprotective role of n-3 PUFAs, notably eicosapentaenoic acid (EPA) and docosahexanoic acid (DHA), referred to as omega-3 fatty acids or fish oil, is supported by substantial evidence. Most observational studies report an inverse relation between fish intake and coronary heart disease (CHD) mortality [20-24], especially sudden cardiac death [25-27]. This protective effect has been attributed to high EPA and DHA blood concentrations [25,28].

More direct evidence of the cardioprotective effect of omega-3 fatty acids comes from RCTs in patients with a history of myocardial infarction (MI). In 2033 post-MI men, a modest intake of fatty fish (200-400 g/week) or of n-3 PUFAs (0.5 g/day) reduced total mortality (primarily CHD deaths) by 29% during the two years of follow-up [29]. By far the largest trial was the GISSI Prevenzione study which tested the efficacy of 1 g/day of omega 3 fatty acids (as EPA and DHA) in 11323 patients with a recent MI (≤3 months) [17]. After 3.5 years, those receiving omega-3 fatty acids had a 20% lower relative risk of total mortality. Sudden death was the most affected cause, with 45% reduction, and the benefit appeared after only four months of therapy [18].

These results might be due to an antiharrythmic action of n-3 PUFAs and are in agreement with evidence from *in vitro *studies [30], animal models [31] and human studies [32].

The antiarrhythmic effect of n-3 PUFAs seems mainly to be due to electrical stabilization of cardiomyocytes, by increasing the electrical stimulus required to elicit an action potential and prolonging the relative refractory time. This might be through an action on sarcolemmal ionic channels: n-3 PUFAs inhibit fast voltage-dependent Na^+ ^currents, L-type calcium currents, and some potassium current components [33].

n-3 PUFAs may also exert beneficial effects through mechanisms such as antithrombotic, antiinflammatory, anti-atherogenic and triglyceride lowering actions, although these have been reported with higher doses than those needed to prevent arrhythmias in clinical settings (1 g/day n-3 PUFAs). These data underlie the American Heart Association recommendations that patients with CHD should consume 1 g/day of EPA+ DHA [34].

## Methods

### Study design and population

**Figure 1:**
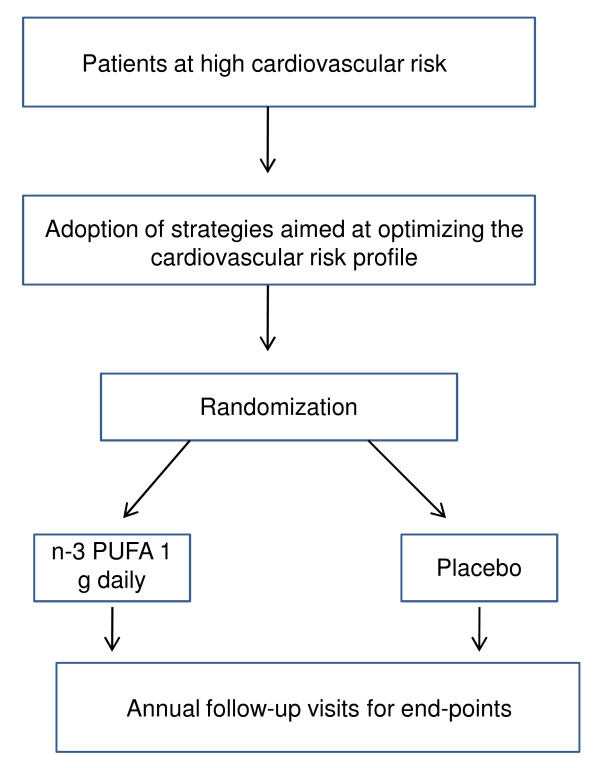
**Rischio & Prevenzione study design**. n-3PUFA indicate polyunsaturated fatty acids.

The R&P study is an ongoing large-scale, multicenter, randomized, double-blind, placebo-controlled clinical trial conducted in the setting of Italian general practice. The primary, experimental, aim of R&P is to assess the efficacy and safety of n-3 PUFAs in reducing cardiovascular mortality (including sudden death) and hospitalization for cardiovascular reasons in patients at high CVD risk but with no history of MI. The secondary, epidemiological, aim is to assess the feasibility of adopting current guidelines in everyday clinical practice in order to optimize all available preventive strategies in people at high cardiovascular risk (Figure 1).

Eligibility criteria were one of the following: 1) clinical evidence of atherosclerotic CVD such as angina pectoris, peripheral artery disease, previous ischemic stroke, transient ischemic attack or revascularization procedure; 2) multiple risk factors (at least four CV risk factors in non-diabetic patients and one or more in diabetics) including: old age (≥ 65 years), male sex, history of arterial hypertension, history of hypercholesterolemia, smoking, obesity (BMI ≥30 kg/m^2^), family history of premature CVD (<55 years in father or brother; <65 years in mother or sister); 3) other conditions putting the patient at high cardiovascular risk according to the general practitioner (GP)'s judgement.

The exclusion criteria were: prior MI, allergy to n-3 PUFAs, pregnancy, diseases with predictable poor short-term prognosis, and foreseeable psychological or logistic difficulties that would affect compliance with the project.

### Intervention strategies

Patients were randomly allocated to receive one capsule daily of 1 g n-3 PUFAs (850-882 mg EPA and DHA as ethyl esters with an average ratio of 1.0 to 1.2) or placebo (olive oil).

At the beginning and during the follow-up all patients were also evaluated and treated by their GPs according to current guidelines in order to optimize their CV risk management. At each visit GPs discussed with the patients their level of CV risk and the possible therapeutic strategies to be adopted or improved (e.g., control of blood pressure, glucose or cholesterol levels, weight reduction, stop smoking, increase physical activity, choose healthier foods, prescription of antiplatelets, etc.), and the therapeutic targets to be achieved in the next study period, completing an *ad hoc *check list.

### Study procedures

GPs were selected either through participation in previous research projects [16,35] or through the investigators' registries at local health units. GPs were invited to attend training meetings held with the patronage of local health units. During these meeting guidelines on cardiovascular prevention were presented and discussed.

Eligible patients were informed and gave their written consent to the study. Treatment was centrally allocated by telephone according to a computer-generated randomization list stratified by GP.

### Data collection and follow-up

Patients are followed by their GPs according to their usual practice, no additional instrumental/laboratory tests are required for the study. Data collection is scheduled annually, and follow-up is planned to last five years.

At enrolment, height, weight, waist circumference, heart rate and blood pressure were measured. History of CVD, risk factors and information on demographic factors, social status, lifestyle habits, laboratory tests and current medical therapies were collected. Patients were also required to complete a simple questionnaire about dietary habits, physical activity during leisure time, social life, psychological status and perceived CV risk level.

Clinical outcomes and adverse events are recorded when they advise and notified with an *ad hoc *clinical form.

Follow-up clinical visits include checking compliance and tolerance to the treatment. Updated information on any new diagnosis of CVD, control of risk factors, changes in lifestyle or dietary habits, and use of medications is also collected

All patients are to be followed up to the scheduled end of study, regardless of adherence to or discontinuation of the treatment for any reason. Study treatments are supplied according to the local health units' procedures.

### Efficacy

The main endpoint of the trial testing the efficacy of n-3 PUFA treatments is cardiovascular death or hospitalization for cardiovascular reasons. Secondary efficacy measures include: 1) the cumulative rate of death, non-fatal MI, non-fatal stroke; 2) the cumulative rate of cardiovascular death, non-fatal MI, non-fatal stroke; 3) coronary deaths; 4) sudden deaths. Additional analysis will be done on each component of the primary endpoint and in specific subgroups of patients corresponding to different risk levels or comorbidity. A specific evaluation is also planned of the benefit of the treatment on the reduction of fatal and non-fatal coronary and arrhythmic events. All outcome events are adjudicated by an *ad hoc *committee blind to the treatment allocation on the basis of pre-agreed definitions and procedures. All reports include a narrative summary and supporting documentation for every event (e.g. clinical records, death certificates and any other relevant documentation).

To assess the feasibility of optimizing cardiovascular prevention in the study population, the preventive strategies adopted and the goals chosen at the outset and during follow-up will be analyzed, as well as their efficacy in terms of risk factor control and therapeutic targets attained.

### Safety

All serious adverse events (defined as fatal, life-threatening, requiring or prolonging hospitalization, permanently disabling or incapacitating, which may jeopardize the subject or which may require medical or surgical intervention), not necessarily drug-related, are reported on a standard form and communicated to the study Coordinating Center within 24 hour of notification of their occurrence. Safety is monitored by an external Data Safety Monitoring Board (DSMB).

### Statistical aspects

The primary endpoint at the start of the study was the cumulative rate of death plus non-fatal-MI and non-fatal stroke. The expected incidence of the primary endpoint was based on the literature and on the events observed in a subgroup of patients of a previous trial conducted in a similar setting of patients and GPs (16). Overall, the rate of events included in the primary endpoint was expected to be 2% per year (10% in five years). The goodness of these sample size calculation was to be assessed after one year of follow-up and the observed rate of events was in fact much lower than expected (1.4% instead of 2%).

Because n-3 PUFA seemed to have a positive influence on various CV outcomes besides the MI and stroke, and considering the increasing burden for public health systems of CV events requiring hospitalization, time to first occurrence of CV death and hospitalization for CV reason was then established as the primary efficacy endpoint of the trial. The expected cumulative rate of these combined end points during the five-year follow-up was assumed to be 15%. The minimum clinically relevant beneficial effect of the test treatment on the cumulative endpoint is set at 15% relative risk reduction (2.25% absolute risk reduction). Accordingly, to detect the efficacy of treatment with a power of 90% and α = 0.05 at least 11,202 patients must be included with a mean follow-up of five years and ≤10% drop outs. To guarantee adequate study power, an event-driven approach was adopted and patients will be followed-up until 1,383 events occur for the primary endpoint.

Statistical differences in the baseline characteristics of the treatment groups will be assessed with the χ^2 ^test for categorical variables and *t *test for continuous variables. Treatment efficacy on the primary endpoint, and on each single component, will be assessed using a Cox's proportional hazards' model [36], and the results will be expressed as hazard ratio (HR) estimates with 95% confidence intervals (CIs). Efficacy analyses will be adjusted for covariates found to be unbalanced between randomised treatments. Kaplan-Meyer estimates of the survival curves will be presented [37]. Time of occurrence of primary combined endpoint will be calculated according to the following hierarchy: time of occurrence of cardiovascular mortality or time of first occurrence of hospitalization for CV reasons.

An interim analysis to assess the efficacy on the primary endpoint is scheduled at approximately half the expected events. This will be monitored using the sequential procedure of Peto [38] and the study will be stopped when the corresponding significance level of evidence reaches α ≤ 0.001.

All analyses will be according to the intention-to-treat approach in which all randomized patients are included, regardless of the adherence to study treatment. A per protocol analysis will be done for patients who take at least 80% of their treatment.

### Ethical/regulatory aspects

The ethical conduct of the study is regulated by the last revision of the Helsinki Declaration. The study is consistent with Good Clinical Practice (GCP) principles and procedures, as described in the ICH Harmonized Tripartite Guideline for Good Clinical Practice (1996); Directive 93/39/CEE The Rules Governing Medicinal Products in the European Community. The study complies with Italian law, Directive 10 May 2001, "Controlled Clinical Trials in the Setting of General Practice and Pediatrics".

The study protocol was approved by the Italian health authorities, the Ethics Committee of the Chairman's Institution and by the local Ethics Committees. An *ad hoc *insurance policy was stipulated for patients and GPs.

This study is registered in a public clinical trial database (ClinicalTrials.gov NCT00317707).

### Trial update

**Figure 2:**
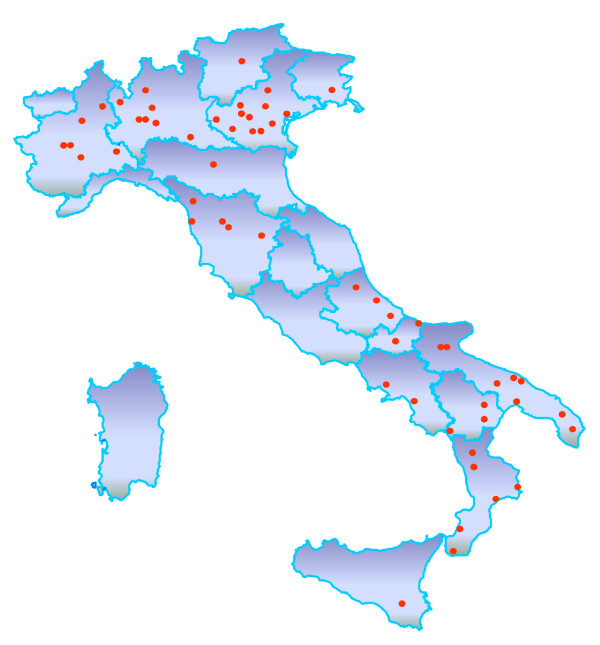
**Rischio & Prevenzione research network**. Red circle = Local health units in the Italian regions participating in the study. The nation-wide research network comprised 860 general practitioners in 57 local health units, who admitted 12,513 patients to the study.

A total of 1,620 GPs attended 34 local training meetings organized throughout Italy; 860 (58%) out of 1486 GPs who initially agreed to join the study started to recruit patients (Figure 2). Between February 2004 and March 2007, 12,513 patients were enrolled. Each GP admitted an average of 14 patients (range 1-79); respectively 271 (31.5%), 564 (65.6%), and 25 (2.9%) GPs recruited ≤10, 11-20, and more than 20 patients.

The main inclusion criterion was diabetes mellitus plus one or more CV risk factors (46.9%). About 30% of patients were admitted because of clinical evidence of atherosclerotic CV, except a history of MI: angina pectoris (12.0%), revascularization procedures (8.8%), peripheral artery disease (7.8%), previous transient ischemic attack (8.1%) or ischemic stroke (4.0%). Multiple CV risk factors (≥4 risk factors, except diabetes mellitus) were the inclusion criterion for 21.1% of patients. Less than 1% were included for other reasons. The main baseline features of the study population are shown in Table 1.

**Table 1:** Baseline characteristics of Risk & Prevention participants

	**% or mean (SD)**
	
*Age (years)*	
Mean	63.9 (9.5)
≤50	7.4
51-59	22.9
60-69	39.6
70-79	27.0
≥80	3.0
	
*Males*	61.5
	
*Previous CVD*	29.6
	
*Cardiovascular risk factors*	
Hypertension	84.6
Hypercholesterolemia	71.1
Diabetes mellitus	59.9
Obesity	48.6
Family history of premature CVD	31.1
Cigarette smokers	21.7
Unhealthy diet	63.9
Physical inactivity	56.1
BMI (kg/m2)	29.3 (4.9)
SBP (mmHg)	140.2 (15.2)
DBP (mmHg)	82.7 (8.2)
Heart rate (bpm)	74.4 (8.4)
Total cholesterol (mg/dL)	215.9 (42.6)
LDL cholesterol (mg/dL)	132.1 (36.4)
HDL cholesterol (mg/dL)	51.1 (13.3)
Triglycerides (mg/dL)	168.7 (99.8)
Fasting blood glucose (mg/dL)	132.8 (46.7)
* HbA1C (%)	7.0 (1.5)
	
*Medical treatment*	
ACE inhibitors	45.1
ARBs	21.9
Diuretics	41.5
Calcium - channel blockers	28.2
β-blockers	20.6
Oral hypoglycemic drugs	44.1
Insulin	6.6
Statins	41.1
Antiplatelet agents	41.4

*in diabetic patients

CVD, cardiovascular disease; BMI, body mass index; SBP, systolic blood pressure; DBP, diastolic blood pressure; LDL, low-density lipoprotein, HDL, high-density lipoprotein, HbA1C, glycated hemoglobin, ACE, angiotensin-converting enzyme; ARB, angiotensin receptor blocker

## Discussion

As shown in Figure 2 and Table 1, the first and certainly one of the most important results of the study is that it proved feasible to create a network of collaborating physicians to generate a prospective cohort adequately representative of practice throughout the country. The GPs' participation is mainly motivated by the acknowledgment that the study is an expression of the duty to continuous professional education. It is also a reminder of the close links between research and practice: the only way to be informed and responsible about whatever is uncertain or unknown, is to investigate it through a research protocol.

The protocol therefore - as a general design, but even more as an operational trial - is the product of a truly collective interplay among dozens of colleagues, and it was open to discussion at the 34 presentations organized throughout the country. One of the GCP requirements - continued education meetings for investigators to verify quality and surveillance of data collection - has been used as an opportunity for sharing and optimizing a project, more than just a teaching exercise on the technicalities of trial procedures.

Consistently with this approach, the inclusion criteria in the trial are not just a list of "objective" data: they mimic the process of an informed decision, which is a mix of documented knowledge, of "perceived" overall clinical condition of risk, and a judgment of opportunity. The epidemiological variability of the study population is not only expected (as explicitly set out in the protocol), but needed, to ensure the population is really representative, and therefore the descriptive and experimental results once they become available can be transferred appropriately.

The patients'actual level of CV risk is lower than expected. However, this is not surprising: beside the fact that the cohort is younger than expected, current patients are more widely exposed to preventive measures. In fact, the rate of events reported in previous or observational studies reflects clinical practice at that time and overestimates the current risk.

The decision to modify the study endpoint fits well with the public health-oriented design of this GP based project and shows that a trial is simply one part of a strategy of comprehensive prospective monitoring and assessment of all those patients deemed to be at a specific risk. Their epidemiological outcomes over the medium-long term provide the most important knowledge from which to interpret the experimental results, which will show whether or not the "new" treatment adds a specific advantage to the "natural" history of this population (i.e. under the GPs' routine care).

Finally, the scientific interest and "modernity" of the experimental hypothesis tested in R&P is confirmed by the GISSI-HF trial results which further support the benefit of n-3 PUFA on mortality seen in GISSI-Prevenzione [17], confirming a significant reduction of deaths in patients with heart failure [39]. Similarly, the benefit of n-3 PUFA in Japanese people suggests that it might be replicated in other populations, and might be even more substantial with higher levels of intake [40]. Novel results like this in the field of cardiology fuel the hypothesis of anti-arrhythmic and anti-atherosclerotic effects of n-3 PUFA and show how important it is to testing their efficacy in a GP's population of patients at high CV risk.

## Abbreviations

CHD: Coronary Heart Disease; CIs: Confidence Intervals; CVD: Cardiovascular Disease; CV: Cardiovascular; DHA: Docosahexanoic acid; DSMB: Data Safety Monitoring Board; EPA: Eicosapentaenoic acid; GCP: Good Clinical Practice; GISSI-HF: Gruppo Italiano per lo Studio della Sopravvivenza nell'Infarto miocardico - Heart Failure; GISSI-Prevenzione: Gruppo Italiano per lo Studio della Sopravvivenza nell'Infarto miocardico-Prevenzione; GP: General Practitioner; HR: Hazard Ratio; MI: Myocardial Infarction; N-3 PUFA: n-3 polyunsaturated fatty acids; RCTs: Randomized Controlled Trials; R & P: Rischio & Prevenzione.

## Competing interests

RM received research support or honoraria for lectures from SPA, Pfizer Sigma-Tau, Solvay, and Ferrer. FA, SB, MCR, MGS and GT received research support from SPA, Pfizer and Sigma-Tau. VC, PL and MT declare that they have no conflict of interest.

## Authors' contributions

This protocol is the result of a collaborative effort which involved the entire research network of GPs with the collaboration of the investigators who are dedicated to the study conduction and management (see appendix). FA, VC, PL, RM, MCR, MGS, GT and MT contributed to the conception and design of the protocol and to draft the manuscript. SB is responsible for statistical and data analysis. All authors will participate in the interpretation of results. The final version of the paper has been revised and approved by the members of the Steering Committee and of the Scientific and Organizing Secretariat.
